# A combination of transposable elements and magnetic cell sorting provides a very efficient transgenesis system for chicken primary erythroid progenitors

**DOI:** 10.1186/1472-6750-9-81

**Published:** 2009-09-18

**Authors:** Camila Mejia-Pous, José Viñuelas, Claudine Faure, Joanna Koszela, Koichi Kawakami, Yoshiko Takahashi, Olivier Gandrillon

**Affiliations:** 1 Equipe "Bases Moléculaires de l'Autorenouvellement et de ses Altérations", Université de Lyon, F-69622, Université Lyon 1, Villeurbanne, CNRS, UMR5534, Centre de Génétique Moléculaire et Cellulaire, Lyon, France; 2 Division of Molecular and Developmental Biology, National Institute of Genetics, and Department of Genetics, The Graduate University of Advanced Studies (SOKENDAI), Mishima, Shizuoka 411-8540, Japan; 3 Nara Institute of Science and Technology (NAIST) 8916-5, Takayama, Ikoma, NARA, 630-0192, Japan

## Abstract

**Background:**

Stable transgenesis is an undeniable key to understanding any genetic system. Retrovirus-based insertional strategies, which feature several technical challenges when they are used, are often limited to one particular species, and even sometimes to a particular cell type as the infection depends on certain cellular receptors. A universal-like system, which would allow both stable transgene expression independent of the cell type and an efficient sorting of transfected cells, is required when handling cellular models that are incompatible with retroviral strategies.

**Results:**

We report here on the combination of a stable insertional transgenesis technique, based on the Tol2 transposon system together with the magnetic cell sorting (MACS) technique, which allows specific selection of cells carrying the transgene in an efficient, reliable and rapid way.

**Conclusion:**

This new Tol2/MACS system leads to stable expression in a culture of primary chicken erythroid cells highly enriched in cells expressing the transgene of interest. This system could be used in a wide variety of vertebrate species.

## Background

Transient and stable transgenesis is a powerful and fundamental technique for understanding any genetic system. In particular, integration in the genome, which allows for the stable expression of a transgene, is often a prerequisite for studying the biological function of a gene.

Retrovirus-based insertional strategies, the technique most frequently used to obtain stable transgenesis, present some technical drawbacks. First, following insertion, the presence of viral sequences can lead to the trans-activation of nearby cellular genes through the action of strong enhancer/promoter elements contained, for example, within the viral long terminal repeat (LTR) [1-4]. Also, the handling and modification of retroviral vectors is laborious and time-consuming. Finally, since these strategies are based on an infection process, they depend on cellular receptors [5] so their use is limited to a particular species, and sometimes even to a particular cell type. This latter restriction is perhaps the main one when handling a particular cell type or samples from a particular (less commonly used) species, incompatible with retroviral strategies.

**Figure 1:**
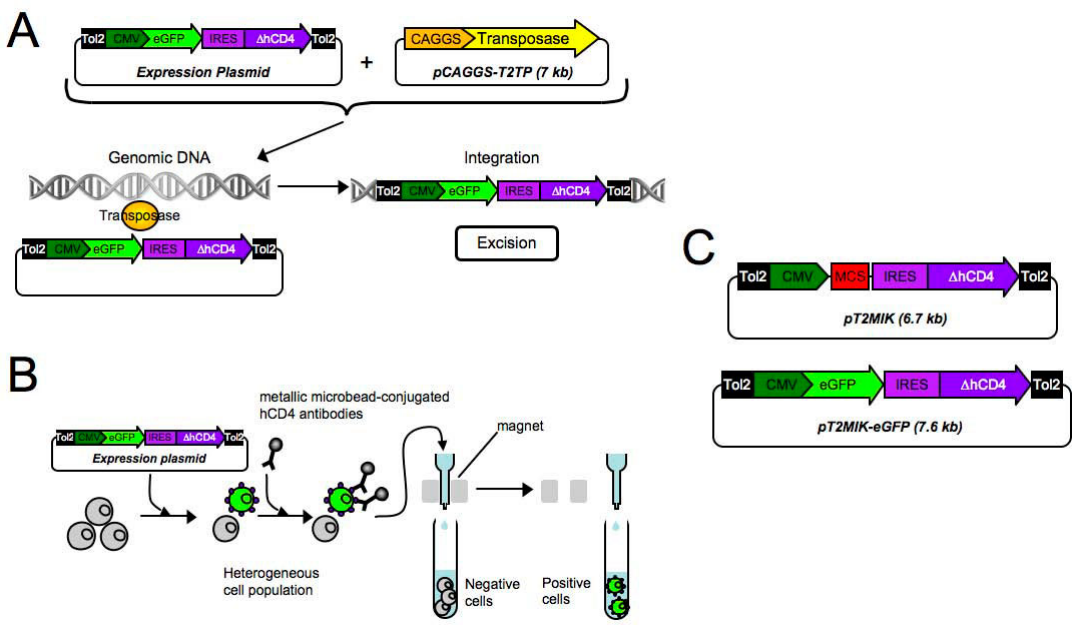
**Stable Transgenesis and Sorting Systems**. **A**: Tol2 transposition system: Cells are cotransfected with the plasmid carrying the Tol2 construct (Expression plasmid) and the helper plasmid, allowing a transient transposase expression (pCAGGS-T2TP). On the one hand, the Tol2 construct contains the expression cassette that should be integrated in the cellular genome, flanked by two Tol2 cis-sequences. On the other hand, the helper plasmid contains the transposase cDNA driven by a CAGGS promoter (a CMV-based constitutive promoter). The Tol2 construct is transposed by a cut-and-paste mechanism that requires the sequence-specific binding of the transposase to the Tol2 sequences on each extremity of the cassette. This transposition process involves the precise excision from the plasmid and integration of the cassette into the genomic DNA. **B**: Magnetic Cell Sorting (MACS): Cells are transfected with a plasmid construct harboring both the transgene and the ΔhCD4-coding sequence. The ΔhCD4 is a surface marker, derived from the human CD4 in which the cytoplasmic region was deleted. Positive cells, expressing both transgene and the ΔhCD4 marker, are magnetically labeled with an antibody, coupled to metallic microbeads, which recognizes the hCD4 extracellular region. After passing the heterogeneous cell population through a column placed on a magnetic field, labeled cells are retained by the column, whereas negative cells pass through. Positive cells are then easily eluted by just moving the column away from the magnet. **C: **The engineered pT2MIK empty plasmid with a multicloning site (MCS) and the pT2MIK-eGFP plasmid used throughout this study.

To circumvent these problems, a novel method of transgenesis, using a transposon-mediated gene transfer technique, has been recently described (see references below). Transposons are genetic elements that can move from one locus in the genome to another and they have been used as powerful tools in model animals and plants. The Tol2 transposable element, which was originally found in the medaka fish [6], has been reported to be capable of undergoing efficient transposition in a wide variety of vertebrate species including zebrafish, frog, mice, chicken and human [7-12]. Recently, cis-sequences necessary for the Tol2 element transposition were revealed [13] which led to the development of transposon vectors containing minimal DNA sequences that are easily manipulated. A plasmid, harboring a Tol2 construct with the gene expression cassette flanked by Tol2 cis-sequences, and a helper plasmid, containing the transposase-coding sequence driven by a constitutive promoter, are simultaneously introduced into vertebrate cells. As a result of the transposase activity, the transposon construct is excised from the plasmid, and subsequently integrated into the host genome ([14], see also Figure 1A).

As the use of the chicken model expands [15], efficient and stable transgenesis methods in this species are increasingly required for many experimental strategies, including genetic screens and dissection of transcriptional regulatory networks. In addition to being a remarkably important and historic model system in developmental biology, genomic resources for *Gallus gallus*, including the genomic sequence [16], have been expanding rapidly. Moreover, *G. gallus *is an interesting source of primary cells for large-scale *ex-vivo *studies, such as the normal erythroid progenitors T2EC (called for TGF-α/TGF-β-induced Erythrocytic Cells) [17]. A transcriptomic approach in T2EC has allowed for the identification of a group of candidate genes important for normal or pathological self-renewal [18-20]. Downstream of these genomic approaches, it is now essential to be able to perform functional studies targeting those candidate genes in order to understand their biological role, for example in the self-renewal process, by acquiring a stable expression of their corresponding cDNAs in a significant proportion of cells among the total population.

We describe here the use of a Tol2-mediated stable gene transfer in primary chicken erythroid progenitors. In order to obtain a significant proportion of cells expressing the gene of interest within the total population, we have associated this Tol2-based transgenesis with the MACS (MAgnetic Cell Sorting) system ([21], see also Figure 1B). We demonstrate that the resulting Tol2/MACS system allows the enrichment of the cell population in cells that stably express the transgene, reported here by the eGFP.

## Methods

### Plasmid constructs

**pT2MIK **(6.7 kb, Figure 1C): pT2AL200R150G [13] was digested with *Xho*I/*Bgl*II to remove the cassette containing the EF1α promoter-eGFP-polyA sequence. These sites were blunt-ended, and the blunt-ended fragment CMV-MCS-IRES-ΔhCD4-polyA, prepared by *Nru*I/*Xho*I digestion from pMACS-4-IRES.II [21], was inserted. The pT2MIK plasmid obtained permits the expression of a bicistronic mRNA controlled by a constitutive promoter (CMV). It harbors a MCS to clone the gene of interest, followed by the ΔhCD4-coding sequence, which is downstream of an IRES to allow its translation.

**pT2MIK-eGFP **(7.6 kb, Figure 1C): the pT2MIK plasmid was digested with *Eco*RV, a site present in the MCS. A blunt-ended *Cla*I fragment from pRCAS.C-EGFP (kindly donated by Pr. Germain Gillet), containing the eGFP coding sequence, was then inserted.

**pCAGGS-T2TP **(7 kb, Figure 1A) [10]: transposase supplier plasmid that contains the transposase cDNA driven by the strong CAGGS promoter (a modified CMV). A transient supply of the CAGGS-controlled transposase appears to be sufficient for the transposition of a transfected gene, as shown below. Moreover, it is assumed that the transient activity of transposase avoids an unnecessary repetition of transposition once the cassette has been integrated into the genome.

**pT2.CMV-hKO **(5.1 kb, see Additional File 1): pT2AL200R150G [13] was digested with *Xho*I/*Bam*HI to remove the cassette containing the EF1α promoter. These sites were blunt-ended, and the blunt-ended fragment containing the CMV promoter, prepared by *Hind*III/*Xba*I digestion from the pCRNCM plasmid [22], was then inserted. The eGFP fragment was subsequently removed by *Cla*I digestion, and a *Cla*I fragment containing hKO, previously extracted from phKO1-S1 (MBL International Corporation) by a *Bam*HI/*Hind*III digestion, was inserted.

### Primary cell culture

T2EC cells were generated from SPAFAS white leghorn chickens (PA12 line from INRA, Tours, France) as previously described [17]. These cells were expanded in LMI medium (α-MEM medium, 10% FBS (Fetal Bovine Serum), 1 mM Hepes, 100 nM β-mercaptoethanol, 1 mM dexamethasone, 5 ng/ml TGF-α, 1 ng/ml TGF-β1, 100 U/ml penicillin and 100 μg/ml streptomycin).

### Cell transfection by nucleofection

Transient transfections were performed by the nucleofection method (Amaxa Technology). For each nucleofection assay, 10^7 ^cells were washed once with PBS and resuspended in 100 μl of nucleofector buffer (Cell line Nucleofector kit V, Amaxa/Lonza). After adding 10 μg of DNA (pT2MIK or pT2MIK-eGFP +/- pCAGGS-T2TP in variable molecular ratios), cells were transferred to the cuvette supplied and nucleofected with the T-16 program of the Nucleofector device (Amaxa/Lonza). Following nucleofection, cells were immediately transferred to LM2 medium (LM1 medium with RPMI 1640 instead of α-MEM) and grown overnight (18 hours maximum).

### Magnetic Cell Sorting (MACS)

To increase the MACS efficiency, dead cells and fragments were removed before each MACS assay. For this, living cells were purified by centrifugation through a density gradient on LSM (Lymphocytes Separation Medium, Eurobio) to get rid of any dead cells and cellular fragments. Up to 10^7 ^cells were resuspended in 1 ml of LM1 and carefully laid onto 1 ml of LSM. After a 15 minute centrifugation at 2500 rpm without brake, the cells on top of the LSM were recovered, washed with 3 ml of PBSA (PBS, BSA 0.5%) and resuspended in PBSA.

Transfected cells, co-expressing the ΔhCD4 marker on the cell surface (Figure 1B), were then magnetically isolated and separated from untransfected cells using the MACSelect - Transfected Cell Selection System. Up to 10^7 ^cells were magnetically labeled with the MACSelect MicroBeads and separated on a MACS Column and an OctoMACS Separator by magnetic force. The sorting was performed according to the manufacturer's instructions, with the following modification: PBSA (PBS, BSA 0.5%) was used instead of PBE (PBS, EDTA 5 mM).

### Immunofluorescent hCD4 staining

10^6 ^cells were labeled with a human CD4 antibody conjugated to allophycocyanin (APC) fluorochrome (CD4-APC Miltenyi Biotec) according to the manufacturer's instructions, with the following modification: no EDTA was added to the staining buffer.

### Flow cytometry analysis

Fluorescence data was acquired with a FACSCanto II flow cytometer and the data was analyzed using the 5.0.1 Diva software (both from Becton-Dickinson). The positive fluorescence threshold was fixed in order to have 99% of the negative cells below this threshold.

### Statistical Analysis

Firstly, in order to determine whether performing a parametric test was possible or not, the homoscedasticity of the distribution (homogeneity of variance) was tested using a Fischer-based test (O'Brien test). In our case, as values were homoskedastic for all the comparisons, only parametrical tests were then used. When comparing only one pair, a Student test (t-test) was applied. When comparing simultaneously three conditions or more, significant differences among the compared conditions were first tested by an ANOVA (Analysis of Variance). For a significant ANOVA test, all pairs' comparison was tested using the Tukey-Kramer HSD test.

### Genomic insertion sites identification by the splinkerette PCR technique

#### Transfection and stably transfected monoclonal T2EC cells

To identify the T2EC Tol2 genomic insertion site, we used a simplified pT2MIK-derived plasmid (pT2.CMV-hKO) expressing the CoralHue humanized Kusabira-Orange fluorescent protein reporter (MBL International Corporation) driven by the CMV promoter. As described above, 5 × 10^6 ^cells were nucleofected with 5 μg of plasmid DNA (pT2.CMV-hKO/Transposase = 5/1). Eight days after transfection, cells expressing the reporter gene were sorted and individually cloned in conical bottom 96-well microplates using a FACSVantage SE cytometer (Becton-Dickinson). Two clones were grown for 13 days, by which time the clonal population size was 2.8 × 10^5 ^and 4 × 10^5 ^cells, prior to DNA extraction.

#### Genomic DNA extraction, restriction endonuclease digestion and splinkerette adaptor ligation

Each of the two clones was lysed in STE buffer (100 mM NaCl, 1 mM Na_2_-EDTA pH 7.8, 10 mM Tris-HCl pH 8) for 1 hour, at 50°C, in the presence of SDS and Proteinase K (Roche). Genomic DNA (gDNA) was then treated for 20 min at 60°C with RNase A (Sigma-Aldrich) before being purified with 1 volume phenol/chloroform/isoamyl alcohol (25:24:1, v/v/v) (Sigma-Aldrich) and precipitated with 2 volumes of absolute ethanol and 1/9^th ^volume of 3 M NaOAc pH 5.2. Subsequently, 1 μg of gDNA was digested with 1 μl of FastDigest *Tai*I (Fermentas), the recognition site of which is a 4 bp motif, for 16 hours at 65°C. This digestion was performed using the Fermentas 10× FastDigest buffer, in a final volume of 20 μl.

Considering that a 4 bp recognition site will occur roughly every 256 bp, and therefore that 200 ng of *Tai*I-digested gDNA correspond to 1.28 pmol DNA, 200 ng of digested DNA were ligated to a 3× molar excess of the annealed splinkerette adaptor (Splinklong-ACGT+Splinkshort-hairpin) with the T4 DNA ligase (4 U), for 1 hour at 22°C, using the Fermentas Rapid DNA Ligation kit. In order to improve the ligation yield, digested DNA was heated for 5 min at 60°C before being ligated.

Finally, the ligation product was purified with phenol/chloroform/isoamyl alcohol following the same protocol as for the gDNA extraction and using glycogen (Fermentas), at a final concentration of 1 μg/μl, in a total volume of 20 μl.

This procedure was also performed for the pT2.CMV-hKO plasmid and for non-transfected T2EC gDNA to obtain, respectively, positive and negative controls of splinkerette PCR.

#### Adaptor and primers

The splinkerette adaptor and the oligonucleotide primers used for this study were synthesized by Invitrogen and are identified as follows:

Splinklong-ACGT: CGAAGAGTAACCGTTGCTAGGAGAGACCGTGGCTGAATGAGACTGGT GTCGACACTAGTGGACGT.

Splinkshort-hairpin: CCACTAGTGTCGACACCAGTCTCTAATTTTTTTTTTCAAAAAAA.

hKOSp1: AGACCGAGGGCAACATCACCGAGC.

Splink1: CGAAGAGTAACCGTTGCTAGGAGAGACC.

nested-hKOSp2: GTGGAGGACGCCGTGGCCCACTGC.

nested-Splink2: GTGGCTGAATGAGACTGGTGTCGAC.

#### Splinkerette PCR

Two rounds of PCR (PCR1 and nested-PCR2), using primers specific for the reporter transgene hKO (hKOSp1 and nested-hKOSp2) and for the annealed splinkerette adaptor (Splink1 and nested-Splink2), were performed with the AccuPrime *Taq *DNA Polymerase High Fidelity (Invitrogen), in a final volume of 50 μl (AccuPrime PCR Buffer II 1×, 200 nM Forward Primer, 200 nM Reverse Primer, 1 U AccuPrime *Taq *DNA Polymerase). The PCR DNA template was 1/300^th ^of the ligation product for the PCR1 and 1/10^th ^of the PCR1 product for the nested-PCR2.

The two PCR rounds were run in a Biometra TRIO-Thermoblock (Biometra) using the following program: 2 min of initial denaturation at 94°C, followed by 30 cycles of 30 s denaturation at 94°C, and 4 min of annealing-elongation at 68°C.

PCR products were then loaded onto 25 cm × 25 cm 1.5% agar gel and purified using the NucleoSpin Extract II (Macherey-Nagel), according to the manufacturer's instructions.

#### DNA sequencing and analysis

DNA sequencing was performed with an ABI PRISM 3100-Avant Genetic Analyzer, according to the instructions of the BigDye Terminator v1.1 Sequencing kit (Applied Biosystems). The Tol2 insertion sites in T2EC gDNA were identified by alignment with the ENSEMBL database  using the BLASTN algorithm.

## Results

### The Tol2 construct allows for stable transgene expression in primary cells

**Figure 2:**
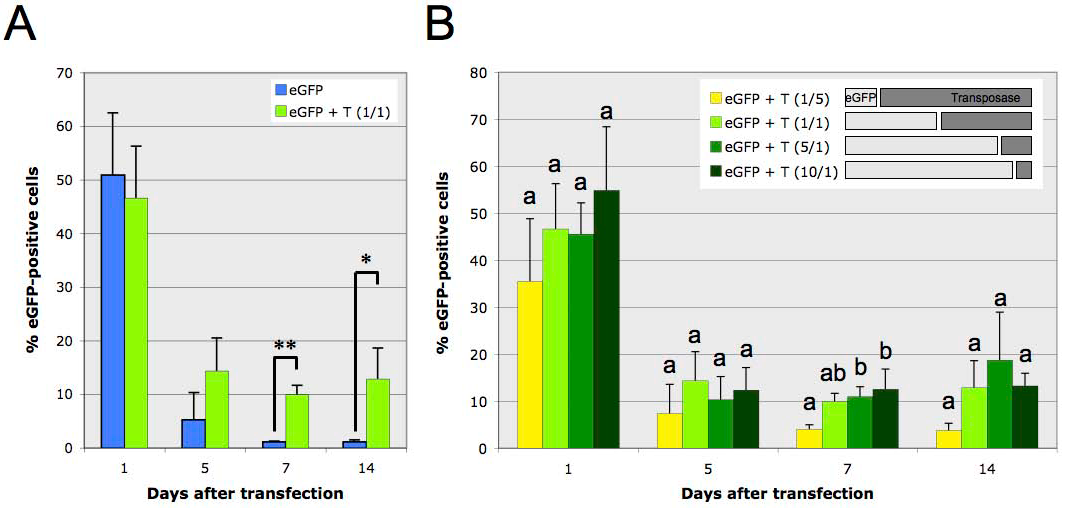
**Stable expression of eGFP in the presence of transposase**. **A**: Relative proportion evolution, with respect to time after transfection, of eGFP-positive cells within a population of T2EC, either transfected with the pT2MIK-eGFP expression plasmid only (eGFP), or cotransfected with the pT2MIK-eGFP plasmid and the transposase expression pCAGGS-T2TP plasmid (eGFP + T) with an equimolar ratio (1/1). Significant differences were determined with a t-Test (n ≥ 3): p = 2.44 × 10^-2 ^(*) and p = 9 × 10^-4 ^(* *). **B**: Relative proportion evolution, with respect to time after transfection, of eGFP-positive cells within a population of T2EC cotransfected with the pT2MIK-eGFP plasmid (eGFP) and the pCAGGS-T2TP plasmid (T). As indicated in the legend, different molecular ratios were tested, pT2MIK-eGFP (7.6 kb)/pCAGGS-T2TP (7 kb): 1/5; 1/1; 5/1 and 10/1, but the amount of total DNA (pT2MIK-eGFP + pCAGGS-T2TP) was constant. For one given time point, conditions not connected by the same letter are significantly different, p < 0.05 (Tukey-Kramer HSD test, n ≥ 3). Cell fluorescence was analyzed by flow cytometry (FACS). The positive fluorescence threshold is fixed in order to have 99% of the negative cells (i.e. cells transfected with the empty plasmid) below this threshold. A 1% value is, hence, considered as null.

We first tested the possibility of generating stable transfectants in T2EC cells using the Tol2 system. For this, T2EC were transfected by nucleofection using the pT2MIK-eGFP plasmid (Figure 1C), either in the absence (eGFP) or in the presence (eGFP+T) of the transposase expression plasmid (Figure 2A), and fluorescence was recorded as a function of time. It was immediately apparent that in the absence of transposase, as expected, no fluorescence was detected one week after transfection. This is in marked contrast to the cells cotransfected with the transposase-supplier plasmid (in equimolar amounts of both plasmids) where 10.0% ± 1.7 of cells within the total population retained eGFP signals for up to 14 days (Figure 2A).

In order to determine optimal cotransfection conditions, we tested different molar ratios of the plasmid carrying the gene of interest and of the helper plasmid. We, thus, observed that the efficiency of stable expression is higher when there is more pT2MIK-eGFP expression plasmid than transposase-supplier helper plasmid (Figure 2B). Indeed, when adding 5 times less eGFP expression plasmid than transposase expression plasmid (1/5 ratio), only 4.0% ± 1.0 of cells expressed eGFP after one week. Equally, we observed that equimolar conditions (1/1 ratio) increased this percentage (i.e. 10.0% ± 1.7), although this difference is not statistically significant. Inversing the ratio, and adding more eGFP expression plasmid than the transposase one, ultimately leads to a significantly higher proportion of eGFP-positive cells: 11,0% ± 2.1 and 12.5% ± 4.3 for 5/1 and 10/1 ratios, respectively. After two weeks, although the differences seem not to be important because of a significant standard deviation (often observed in primary cultures as cells become senescent), the tendency remains the same.

Our data, therefore, demonstrates that the molecular ratio between the Tol2 plasmid and the transposase helper plasmid has an impact on the stable expression efficiency in T2EC. Efficacy is certainly improved when adding higher amounts of the Tol2 construct plasmid than the transposase provider plasmid. The 5/1 ratio leads to an optimal level of expression of the gene of interest, with about 10% of the transfected cells stably expressing the reporter transgene.

### The transgene carried by the Tol2 construct is stably transposed into the cellular genome

**Figure 3:**
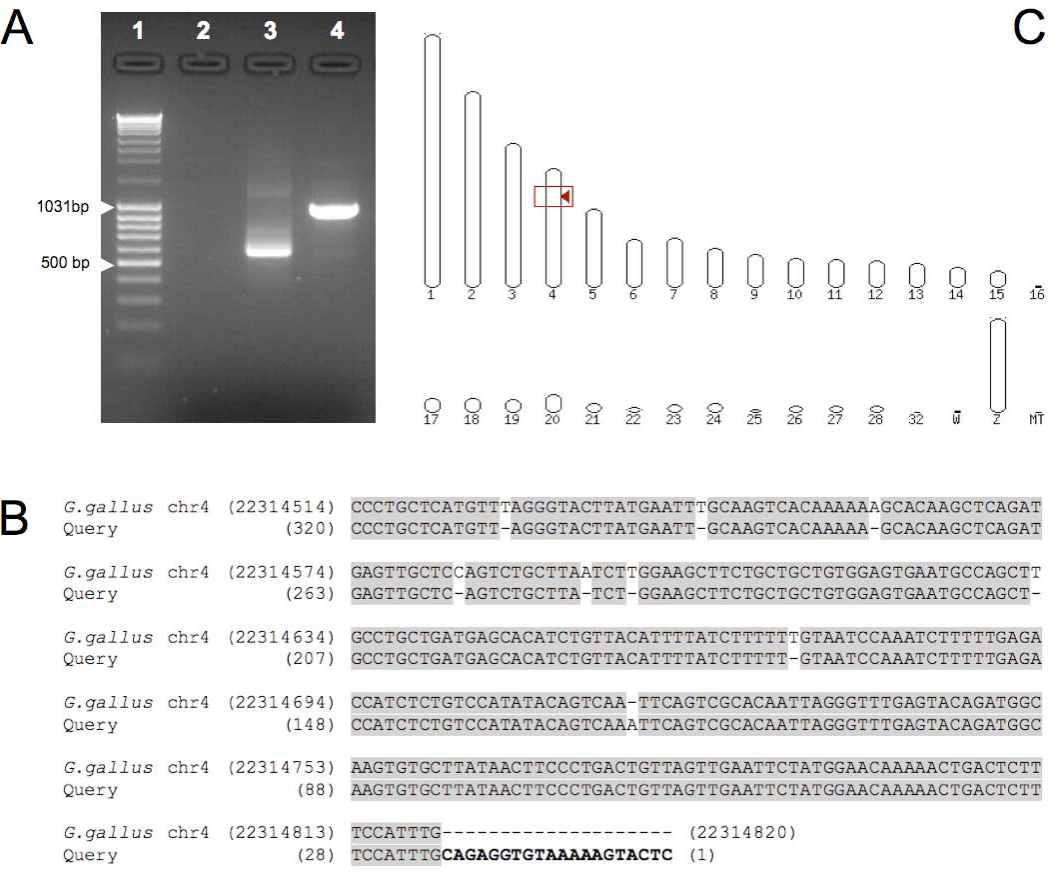
**Identification of the Tol2 construct genomic insertion sites on the T2EC clone #1**. **A**: Splinkerette PCR products on 1.5% agar gel. 1: Fermentas DNA Ladder Mix; 2: genomic DNA from non-transfected T2EC; 3: pT2.CMV-hKO plasmid; 4: genomic DNA from T2EC clone #1, cotransfected with the pT2.CMV-hKO plasmid and the transposase plasmid, pCAGGS-T2TP (molecular ratio: 5/1). **B**: Alignment of the splinkerette PCR product with the *Gallus gallus *genome. The sequence corresponding to the Tol2 construct is shown in **bold**. **C**: Localization of the Tol2 construct insertion site into chromosome 4 of the *Gallus gallus *genome.

Our results strongly suggest that transposition into the genomic DNA (gDNA) was successfully achieved. To confirm this hypothesis, we identified the transgene insertion sites in two T2EC clones transfected with a Tol2 construct, using a modified version of the splinkerette PCR technique [23]. After sequencing the corresponding PCR fragment (Figure 3A) and identifying the Tol2 construct, we aligned all of the "non-Tol2" region sequence with the *G. gallus *gDNA sequence (Figure 3B). We observed that, in a clonal population, there seems to be only one single insertion point, which is located in chromosome 4 (Figure 3C). The second T2EC clone analyzed also shows one single insertion point, localized in a different chromosome (chromosome 1; see Additional File 2).

The cotransfection of the expression plasmid, together with the transposase-supplier helper plasmid, has therefore allowed for the stable expression of the reporter transgene in avian primary erythroid progenitors by transposition of the Tol2 construct into the cellular genome.

### Characterization of the ΔhCD4 surface marker expression

Although 10% of stably positive cells is a sign of efficient transgenesis when working on primary cells, it is still relatively low for studying and observing closely the biological effect of a given transgene. Such a low percentage of cells stably expressing a transgene can be bypassed by clonal selection when working with immortal cell lines. This is clearly not the case when working with normal cells since their lifespan is limited by the so-called "Hayflick limit" [24]. For T2EC cells, which can only be grown for about one month [17], this therefore precludes the use of multiple successive cloning to generate a pure population of cells expressing a transgene of interest. We therefore decided to use the MACS-based method, in which cells carrying the transgene are retained on a magnetic column (Figure 1B).

**Figure 4:**
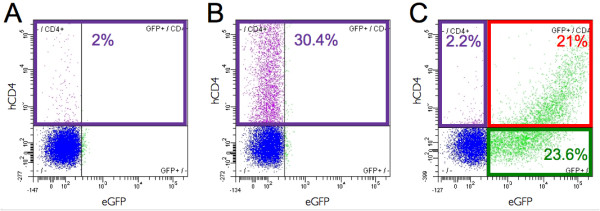
**Quantification of the hCD4 marker and the eGFP expression before MACS**. A population of T2EC not transfected (**A**) or transfected with either the pT2MIK empty plasmid (**B**) or the pT2MIK-eGFP expression plasmid (**C**) were labeled with an APC-conjugated antibody that recognizes the hCD4. Labeled cell fluorescence was analyzed by flow cytometry (FACS) one day after transfection. The positive fluorescence threshold is fixed in order to have 99% of the negative cells (i.e. untransfected and unlabeled cells) below this threshold. A 1% value is, hence, considered as null.

Since the ΔhCD4 sequence is carried by the same bicistronic mRNA as the reporter transgene (Figure 1C), the presence of the ΔhCD4 marker should indicate the presence of the transgene. In order to confirm this feature in our experimental model, ΔhCD4 expression, following different transfection conditions, was quantified using a fluorochrome-conjugated hCD4 antibody (Figure 4). Initially, even though ΔhCD4 is not naturally expressed by T2EC, we observed that a very small percentage of untransfected cells are positive to ΔhCD4 labeling (2%, Figure 4A), suggesting that a small fraction of T2EC expresses a surface protein that cross-reacts with the hCD4 antibody. However, this percentage of false-positive cells is insignificant compared to the ΔhCD4-positive fraction of cells within a population transfected with the pT2MIK empty plasmid (30.4%, Figure 4B) or with the pT2MIK-eGFP expression plasmid (23.2%, Figure 4C). Furthermore, our data shows that among the eGFP-positive cells (44.6% of the total population, Figure 4C), approximately half of them are also ΔhCD4-positive. Indeed, with respect to the total population, 21% of cells are eGFP-positive/ΔhCD4-positive and 23.6% are eGFP-positive/ΔhCD4-negative. If considering the surface marker only, the latter fraction is composed of false-negative cells. Also, as expected, it appears that the ΔhCD4 expression level is correlated to the eGFP expression level and the false-negative cells correspond to the low-level eGFP-expressing cells. Whether these cells express the surface marker at an undetectable level in these conditions or do not express it at all is, as yet, unresolved. Nevertheless, these observations confirm that the pT2MIK-eGFP expression plasmid allows the expression of the ΔhCD4 surface marker, on which the magnetic sorting (MACS) depends.

### MACS allows for the efficient sorting of eGFP-positive cells

**Figure 5:**
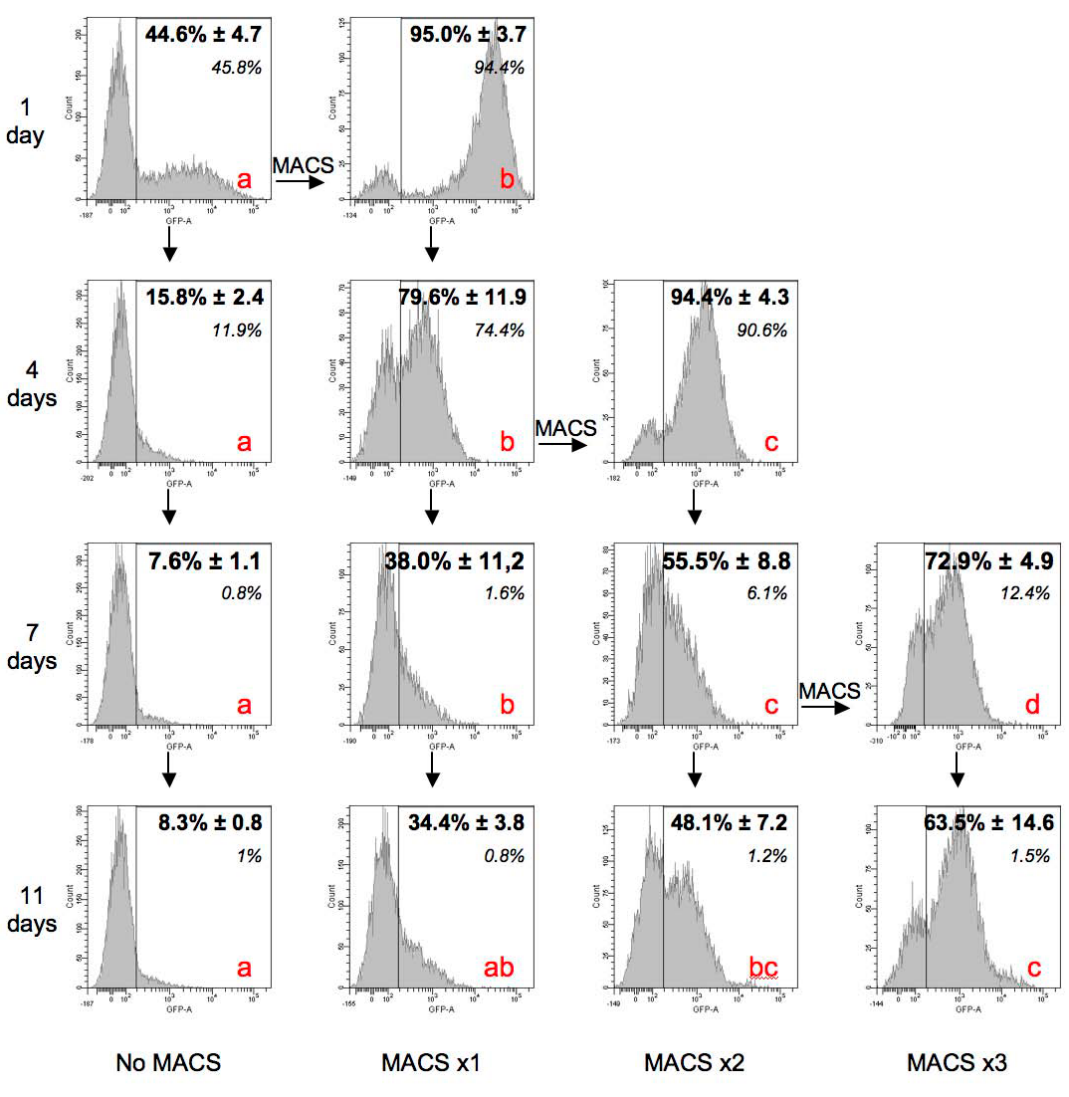
**Enrichment, using MACS, of the transfected population on cells carrying the transgene**. Relative ratio evolution of eGFP-positive cells within a population of T2EC cotransfected with the eGFP expression plasmid and the transposase-supplier helper plasmid (molecular ratio: 5/1). Rows: different time points after transfection (on days), ↓ symbolizes the evolution of the culture as a function of time. Columns: Successive MACS: cell cultures were sorted once (MACSx1), twice (MACSx2), three times (MACSx3) or not sorted at all (no MACS), → symbolizes the evolution of one culture before and after a sorting. Histograms illustrate the cell number repartition according to the fluorescence intensity, from one single representative experiment. Values in **bold **correspond to the mean ± SD after experiment repetition (n = 4). For the same given time point, conditions not connected by the same letter (a, b, c, or d) are significantly different, p < 0.05 (Tukey-Kramer HSD test, n ≥ 3). Values in *italics *correspond to the relative ratio of eGFP-positive cells within a population of T2EC transfected with the eGFP expression plasmid only (no transposase) and sorted once, twice, three times or not sorted at all. Cell fluorescence was analyzed by flow cytometry (FACS). The positive fluorescence threshold is fixed in order to have 99% of the negative cells (i.e. cells transfected with the empty plasmid) below this threshold. A 1% value is, hence, considered as null.

In order to increase the proportion of cells expressing the transgene of interest with respect to the total transfected population, we tested the efficacy of the MACS-based method. As previously observed, in the absence of any selection (Figure 5, first column: No MACS) the relative ratio of cells expressing the eGFP reporter gene dropped rapidly to about 8% and was then stably maintained (7.6% ± 1.1 and 8.3% ± 0.8 for 7 and 11 days, respectively, post-transfection). Following a single round of sorting (Figure 5, second column: MACS x1), the culture already contains a significantly higher rate of cells carrying the transgene in a stable manner. The percentage of eGFP-positive cells, with respect to the total population, is four to five times higher than in unsorted cells (38.0% ± 11.2 and 34.4% ± 3.8 at day 7 and 11, respectively). When the sorting is extended to three successive times, the same culture (Figure 5, fourth column: MACS x3) reaches a relative ratio of about eight to nine times higher than the non-sorted culture (72.9% ± 4.9 and 63.5% ± 14.6 at day 7 and 11, respectively). Although at the end of the experiment the eGFP-positive cell percentage, with respect to the total population, in the MACSx2 condition (Figure 5, third column: 48.1% ± 7.2 after 11 days post-transfection) is not significantly different from both the MACSx1 and the MACSx3 conditions, it is a necessary transitory step towards the three times-sorted culture, in which the positive cell percentage differs significantly from both the non-sorted and the once sorted cultures.

Thus, the MACS-based technique has enabled us to significantly enrich a population of transfected primary progenitor cells with eGFP-positive cells.

### MACS exclusively sorts cells that stably express the transgene

In order to assess whether the eGFP-positive cells sorted after three successive rounds were stably positive cells, we tested the same protocol of successive rounds of MACS on a T2EC population transfected with the eGFP expression plasmid only which, consequently, shows a transient eGFP expression (Figure 5, italic values). On day 4, with respect to the length of time following transfection, we observed that the first two rounds of MACS successfully sorted eGFP-positive cells in the absence of transposase and, hence, those transiently expressing the transgene. This sorting gives a population within which there is a high percentage of eGFP-positive cells (94.4% and 90.6% after one and two MACS, respectively). However, between the fourth and the seventh day which, according to our previous observations, is the period when the transient expression is lost and the stably expressing transfectants emerge (Figure 2A), the percentage of eGFP-positive cells dropped dramatically to 6.1%. Although the third MACS separation moderately increases this percentage (to 12.4%), these transient-expressing cells are rapidly lost at the next point of measurement (1.5%, which virtually overlaps with the 1% null threshold).

Taken together, our data show that the Tol2 transposition system can be associated with a MACS-based technique which significantly improves the stable expression efficiency of the resulting Tol2/MACS system. In fact, this association significantly increases the proportion of cells expressing the transgene among the total transfected population.

## Discussion

We have described an efficient technique which allows the selection of the stable integration of an exogenous transgene into the host genome. The importance of this study is twofold:

1. we have demonstrated that the versatility of the Tol2 system can be extended to normal chicken erythroid progenitor cells and

2. we have demonstrated the feasibility of combining this chromosomal integration technique with a MACS (MAgnetic Cell Sorting)-based enrichment method, allowing the instantaneous sorting of cells carrying the transgene.

The value of the Tol2 transposon-derived transgenesis strategy has been confirmed by reports of its ability to undergo efficient transposition in a wide variety of vertebrate species [7-12]. Among the DNA transposable elements, the Sleeping Beauty transposon, a member of the *mariner *family [25], has been previously reported to be capable of undergoing efficient transposition in chicken [26]. However, a Sleeping Beauty-based transgenesis system was found to be inefficient in primary avian cells, despite its efficacy when tested on immortalized avian cells [27]. The present report demonstrates that Tol2, contrary to Sleeping Beauty, provides an efficient transgenesis system in primary avian erythroid progenitors.

Regarding the optimal molecular ratio between the expression and the helper plasmid, our data suggest that transposition is more efficient when cells are cotransfected with more of the Tol2 expression plasmid than the transposase one. This observation seems to indicate that there is an "overproduction inhibition", which leads to a decrease in transpositional activity in the presence of an increased amount of transposase [28]. Nevertheless, it has recently been reported that the Tol2 transposon-based system does not exhibit overexpression inhibition within the tested transposase concentration range [12]. Even if, at first sight, there seems to be a discrepancy between this report and our results, there is a subtle experimental distinction which may explain this difference. In reference [12], the total amount of DNA was variable. This is in contrast with our own study in which we decided to fix the total DNA amount. The increase in the level of the transposase plasmid was then accompanied by a decrease in the amount of expression plasmid. Moreover, in our cellular model the efficiency of the Tol2/MACS system is proportional to the quantity of Tol2 expression plasmid present, as well as the number of cells to be transfected (see Additional File 3). Therefore, the reason for the decreased transpositional efficacy observed with the 1/5 ratio is likely to be the decrease in the amount of Tol2 expression plasmid rather than an "overexpression inhibition".

In our experimental model, we were limited both by a maximal total of DNA used for the transfection and by the number of cells to be transfected, since the cell viability after nucleofection is inversely proportional to these parameters (see Additional File 3). These features are inherent to our model (i.e. primary erythroid progenitors and the nucleofection technique) so, when transferring the Tol2/MACS method to other cellular models and/or other transfection techniques, this should be set and optimized in order to take full advantage of this method.

We developed an approach based on the splinkerette technique [23] that allowed us not only to prove that there is an integration into the cellular genome, but also to actually characterize the insertion sites. Our results show that there is one single insertion point per T2EC clone analyzed, suggesting that the transposition event is a quite discrete event which probably leads to only one (or at most very few) insertion site. The single insertion point is localized on different chromosomes in the two analyzed clones. Whether this insertion site localization is random or whether there are some preferential genomic regions cannot currently be determined.

In order to obtain a population where a significant proportion (if not all) of the cells express the transgene, it is necessary to select or sort the transfected cells. When working on established cell lines, a common method consists of establishing transgenic clones or lines, which can be time-consuming. Although working *ex-vivo *with primary cells allows for more relevant studies (because they are normal and non-genetically altered cells), the lifespan of such cells is, by definition, limited. Hence we have chosen the MACS technique as it allows instantaneous enrichment of the population of cells carrying the transgene, unlike selection-based techniques which are longer and often introduce a bias due to their high selectivity. For example, it has been reported that selecting antibiotic-based agents can alter cellular metabolism genes [29]. Our data shows that the present MACS-based method leads, after three successive selections, to a population in which about 70% of the cells stably express the transgene. Previous work in the laboratory [30] showed that transitory transfection of *SCA2*, a gene involved in the self-renewal of T2EC, has significant biological effects for up to three days after transfection. As these progenitors have a rapid cycle (they divide every 18 hours [17]), the transgene expression decreases in the whole population. In fact, cells carrying the transgene are diluted in a population that has doubled its size four times in three days. Despite this, three days after *SCA2 *transfection a significant effect can still be observed, which demonstrates that this effect can be detected even if there is only a small fraction of cells expressing it. Therefore, having 70% of cells expressing the transgene in a stable manner within the population is sufficient for carrying out studies on the genetic functions in these cells.

The efficacy of the MACS system relies upon the expression of the cell surface marker ΔhCD4, the translation (and, hence, expression) of which depends on the efficacy of the IRES. It has been shown that IRES efficiency is lower than that of the "classic" ribosome entry site (at the mRNA 5' extremity, which drives the cap-dependent translation) [31]. Characterization of the expression of this marker, compared with the transgene expression reported by the eGFP, confirmed this assumption. Therefore, when using the present method, one should take into account that about half of the positive cells will actually appear as false-negatives (i.e., expressing the transgene but not the marker). Moreover, the expression level of ΔhCD4 is an important parameter, since the efficacy of positive cell retention on the magnetic column depends on the amount of microbead-conjugated antibodies at the cell surface, which is proportional to the expression level of this surface marker. In our experiments, the first MACS selection (leading to almost 100% positive cells) was carried out between 14 and 18 hours after transfection, since we observed that a MACS cell sorting performed more than 24 hours later was remarkably less efficient (data not shown). Timing is, therefore, an important parameter since we observed that the earlier the selection is done, the higher the expression level is and, consequently, the higher the efficiency. According to these observations, it is tempting to argue that the time point we use is so rapid that we are likely not to select stable integration events. However, our data shows that even if the first two MACS rounds of separation are equally efficient on cells expressing the transgene stably or transiently (with or without transposase), the third one is only efficient when cells are cotransfected with transposase and, hence, stably express the transgene. This suggests that these first two MACS selections are needed to increase both the proportion of cells expressing the transgene and the expression level of the ΔhCD4 surface marker in order to increase the efficacy of the third, and last, MACS separation, which is in fact the most decisive.

Furthermore, the fact that cells transfected with the empty plasmid also express the ΔhCD4 marker shows that the absence of a gene cloned upstream of the IRES does not impair its expression. This is an important feature since, in functional studies based on transgene expression, the negative control is often the condition transfected with the same empty plasmid. Therefore, the present strategy allows the sorting of cells transfected with the empty plasmid in the same way as cells transfected with the transgene-containing plasmid and, hence, provides a pertinent minus control. We also observed that, even though it is marginal, there is a small fraction of cells that would appear as false-positives and could, therefore, be selected. This may explain, at least partially, the fact that after each MACS step there is always a fraction of negative cells remaining (not expressing the transgene). However, this could also be explained by the extinction of the transgene expression. As both the IRES efficacy and the specificity of labeling with the microbeads-conjugated antibodies depend on the cell type, this should be tested in a preliminary experiment when transferring the current method to another experimental model.

According to the ΔhCD4 expression data, it is tempting to suppose that FACS (Fluorescence-Activated Cell Sorting ([32]) is much more efficient than MACS, but even if there is a difference in performance it is not particularly striking. Supposing the same method was adapted to FACS (i.e. with a fluorescent reporter gene downstream of the IRES instead of the ΔhCD4), there would also be a fraction of false-negative cells since the level of reporter gene expression depends on the efficiency of the IRES. Therefore, the proportion of true-positive cells (i.e., expressing the transgene and the reporter fluorescent gene) would not be very different from the one observed with the ΔhCD4, which is about 20% in T2EC. In practice, for MACS, we determined in our experimental model that up to 10% of the cells are actually sorted (see Additional File 4) This observation suggests that among the 20% of cells expressing the ΔhCD4, the magnetic column will actually retain only half. FACS is based on optical properties detected by an instrument, whereas MACS depends on several physical interactions (ΔhCD4 marker/antibody/magnetic column), so it is possible that FACS would have a higher sensitivity and this could increase the percentage of sorted cells up to 20%.

Nevertheless, if this constitutes an obvious advantage of FACS over MACS, there are also many advantages of MACS over FACS. First of all, FACS requires the expression of a fluorescent reporter gene, which is generally present in the cytoplasm. The expression of the MACS-required ΔhCD4, which is only at the cell surface without any cytoplasmic region present (as it has been deleted), is therefore more confined and more silent as regards cell function. Also, the antibody-conjugated microbeads are ultrasmall (approx. 50 nm ∅), biodegradable, and non-toxic to cells. Thus, transfected cells are magnetically selected without affecting cell function or viability. Furthermore, from a purely practical point of view, the MACS technique and equipment are more accessible than those used for FACS. In fact, FACS requires the appropriate sorter, an instrument that may not be available in the laboratory. On the other hand, the MACS equipment is very simple: the magnetic sorter can be placed on the lab bench as it is as easy to handle as a normal test tube rack. Moreover, whereas FACS selection can be time-consuming, since it sorts cell by cell, with a MACS experiment it is possible to instantly sort up to 10^7 ^positive cells, within 2 × 10^8 ^cells per column, in about one hour (including the labeling and wash steps), with the column version used in our experiment. For cells which have a reduced viability outside of the incubator, this could make a huge difference. In addition, it is possible to work with several columns at the same time, or to work with high capacity columns (up to 10^8 ^positive-cells within 2 × 10^9 ^cells per column).

However, it is important to note that our system is compatible with a Tol2/FACS variant. Instead of using the microbead-conjugated antibody and a magnetic column, it is possible to use a fluorochrome-conjugated hCD4 antibody (equivalent to the one used to characterize the expression of the ΔhCD4 in this study) to label the cells carrying the transgene and then sort these cells according to their fluorescence.

The MACS technique provides an efficient, rapid, gentle and easy way to sort cells, but methodological improvements could still be made. We believe that since the efficiency of the IRES-based expression might be questionable (see above), the priority is to develop an alternative Tol2/MACS system without the drawbacks of the IRES. We are, therefore, currently designing a Tol2 construct with two genes (the one of interest and the ΔhCD4-coding sequence) driven by two different promoters, leading to higher expression on the ΔhCD4 and, hence, a higher MACS efficiency. However, it is known that constitutive and highly active promoters can mutually interfere with each others functioning [4]. This is why it seems relevant to add, between the transgene and the sorting gene, an insulator [33] to block any interference between the two promoters. The functional analysis of such a construct is, however, beyond the objectives of the current study.

It is, therefore, expected that further improvements can be made to extend the versatility of this stable expression strategy, combined with the simple and efficient MACS method. This strategy will then allow a genetic manipulation of target cells/tissues in a wide range of cell types and species. Moreover, this strategy is perfectly suited for primary cells, which are non-immortalized and often delicate, and, more generally, in all cases when time becomes a critical parameter.

## Conclusion

We have described a new Tol2/MACS selection system that leads to stable expression in a culture of primary chicken erythroid cells highly enriched in cells expressing the transgene of interest. This system could be used in a wide variety of vertebrate species and is perfectly suited for primary cells, which are often delicate, and, more generally, in all cases when time becomes a critical parameter.

## Authors' contributions

CMP performed all the cellular experiments, except the genome insertion point identification, participated in the design and the production of the molecular constructs and drafted the manuscript. JV performed the clonal seeding set-up, the genome insertion point identification experiments and the statistical analyses. CF participated in the design and the production of the molecular constructs. JK performed the transfection experiment demonstrating the proof of the concept of Tol2 usage in T2EC. KK and YT provided the pT2AL200R150G and pCAGGS-T2TP plasmids and provided guidance on their use. KK confirmed the genome insertion point identification using a different technique. OG conceived the study and participated in its design and coordination. All authors read and approved the final manuscript.

## Supplementary Material

Additional file 1 **pT2.CMV-hKO plasmid map**. *Tai*I restriction sites are shown. Click here for file

Additional file 2 **Identification of the Tol2 construct genomic insertion sites on T2EC clone #2**. (**A) **Alignment of the splinkerette PCR product with the *Gallus gallus *genome. The sequence part of the Tol2 construct is shown in **bold**. **(B) **Localization of the Tol2 construct insertion site into chromosome 1 of the *Gallus gallus *genome. Click here for file

Additional file 3 **Influence of the amount of DNA and the cell number on MACS efficiency**. (**A) **Relative proportion evolution, with respect to time after transfection, of eGFP-positive cells within a population of T2EC cotransfected with the eGFP expression pT2MIK-eGFP plasmid and the transposase expression pCAGGS-T2TP plasmid with a 5/1 molecular ratio. Different total DNA (pT2MIK-eGFP+ pCAGGS-T2TP) amounts (5 μg or 10 μg) and cell numbers (5 × 10^6 ^or 10 × 10^6^) were tested. Cell fluorescence was analyzed by flow cytometry (FACS). The positive fluorescence threshold is fixed in order to have 99% of the negative cells (i.e. cells transfected with the empty plasmid) below this threshold. A 1% value is, hence, considered as null. **(B) **Percentage of living cells, for the same conditions as (**A**), according to the morphology characteristics (size and granularity) measured by flow cytometry (FACS) one day after transfection. Click here for file

Additional file 4 **Cell number before and after each MACS step**. A population of T2EC cotransfected with the eGFP expression plasmid and the transposase-supplier helper plasmid (molecular ratio: 1/1) was successively sorted three times on day 1, day 4 and day 7, with respect to time after transfection (Respectively MACSx1 and MACSx2, MACSx3). The cell number was determined before (input) and after (output) each MACS step and the ratio output/input was calculated. Click here for file

## References

[B1] Bartholomew C, Ihle JN (1991). Retroviral insertions 90 kilobases proximal to the Evi-1 myeloid transforming gene activate transcription from the normal promoter. Mol Cell Biol.

[B2] Lazo PA, Lee JS, Tsichlis PN (1990). Long-distance activation of the Myc protooncogene by provirus insertion in Mlvi-1 or Mlvi-4 in rat T-cell lymphomas. Proc Natl Acad Sci USA.

[B3] Morishita K, Parker DS, Mucenski ML, Jenkins NA, Copeland NG, Ihle JN (1988). Retroviral activation of a novel gene encoding a zinc finger protein in IL-3-dependent myeloid leukemia cell lines. Cell.

[B4] Weber EL, Cannon PM (2007). Promoter choice for retroviral vectors: transcriptional strength versus trans-activation potential. Human gene therapy.

[B5] Coffin JM, Fields BN, Howley PM, Knipe DM (1996). Retroviridae: the viruses and their replication. Fundamental virology.

[B6] Koga A, Suzuki M, Inagaki H, Bessho Y, Hori H (1996). Transposable element in fish. Nature.

[B7] Kawakami K, Koga A, Hori H, Shima A (1998). Excision of the tol2 transposable element of the medaka fish, Oryzias latipes, in zebrafish, Danio rerio. Gene.

[B8] Kawakami K, Shima A, Kawakami N (2000). Identification of a functional transposase of the Tol2 element, an Ac-like element from the Japanese medaka fish, and its transposition in the zebrafish germ lineage. Proc Natl Acad Sci USA.

[B9] Kawakami K, Imanaka K, Itoh M, Taira M (2004). Excision of the Tol2 transposable element of the medaka fish Oryzias latipes in Xenopus laevis and Xenopus tropicalis. Gene.

[B10] Kawakami K, Noda T (2004). Transposition of the Tol2 element, an Ac-like element from the Japanese medaka fish Oryzias latipes, in mouse embryonic stem cells. Genetics.

[B11] Sato Y, Kasai T, Nakagawa S, Tanabe K, Watanabe T, Kawakami K, Takahashi Y (2007). Stable integration and conditional expression of electroporated transgenes in chicken embryos. Dev Biol.

[B12] Balciunas D, Wangensteen KJ, Wilber A, Bell J, Geurts A, Sivasubbu S, Wang X, Hackett PB, Largaespada DA, McIvor RS (2006). Harnessing a high cargo-capacity transposon for genetic applications in vertebrates. PLoS genetics.

[B13] Urasaki A, Morvan G, Kawakami K (2006). Functional dissection of the Tol2 transposable element identified the minimal cis-sequence and a highly repetitive sequence in the subterminal region essential for transposition. Genetics.

[B14] Kawakami K (2007). Tol2: a versatile gene transfer vector in vertebrates. Genome Biol.

[B15] Stern CD (2005). The chick; a great model system becomes even greater. Dev Cell.

[B16] Consortium ICGS (2004). Sequence and comparative analysis of the chicken genome provide unique perspectives on vertebrate evolution. Nature.

[B17] Gandrillon O, Schmidt U, Beug H, Samarut J (1999). TGF-beta cooperates with TGF-alpha to induce the self-renewal of normal erythrocytic progenitors: evidence for an autocrine mechanism. Embo J.

[B18] Dazy S, Damiola F, Parisey N, Beug H, Gandrillon O (2003). The MEK-1/ERKs signalling pathway is differentially involved in the self-renewal of early and late avian erythroid progenitor cells. Oncogene.

[B19] Damiola F, Keime C, Gonin-Giraud S, Dazy S, Gandrillon O (2004). Global transcription analysis of immature avian erythrocytic progenitors: from self-renewal to differentiation. Oncogene.

[B20] Bresson C, Keime C, Faure C, Letrillard Y, Barbado M, Sanfilippo S, Benhra N, Gandrillon O, Gonin-Giraud S (2007). Large-scale analysis by SAGE reveals new mechanisms of v-erbA oncogene action. BMC Genomics.

[B21] Gaines P, Wojchowski DM (1999). pIRES-CD4t, a dicistronic expression vector for MACS- or FACS-based selection of transfected cells. Biotechniques.

[B22] de la Pompa JL, Zeller R (1993). Ectopic expression of genes during chicken limb pattern formation using replication defective retroviral vectors. Mech Dev.

[B23] Devon RS, Porteous DJ, Brookes AJ (1995). Splinkerettes--improved vectorettes for greater efficiency in PCR walking. Nucleic Acids Res.

[B24] Hayflick L (1965). The Limited in Vitro Lifetime of Human Diploid Cell Strains. Exp Cell Res.

[B25] Medhora M, Maruyama K, Hartl DL (1991). Molecular and functional analysis of the mariner mutator element Mos1 in Drosophila. Genetics.

[B26] Sherman A, Dawson A, Mather C, Gilhooley H, Li Y, Mitchell R, Finnegan D, Sang H (1998). Transposition of the Drosophila element mariner into the chicken germ line. Nat Biotechnol.

[B27] Kong BW, Carlson DF, Fahrenkrug SC, Foster DN (2008). Application of the Sleeping Beauty transposon system to avian cells. Animal genetics.

[B28] Lohe AR, Hartl DL (1996). Autoregulation of mariner transposase activity by overproduction and dominant-negative complementation. Molecular biology and evolution.

[B29] Rodolosse A, Barbat A, Chantret I, Lacasa M, Brot-Laroche E, Zweibaum A, Rousset M (1998). Selecting agent hygromycin B alters expression of glucose-regulated genes in transfected Caco-2 cells. The American journal of physiology.

[B30] Bresson-Mazet C, Gandrillon O, Gonin-Giraud S (2008). Stem cell antigen 2: a new gene involved in the self-renewal of erythroid progenitors. Cell proliferation.

[B31] Mizuguchi H, Xu Z, Ishii-Watabe A, Uchida E, Hayakawa T (2000). IRES-dependent second gene expression is significantly lower than cap-dependent first gene expression in a bicistronic vector. Mol Ther.

[B32] Hulett HR, Bonner WA, Barrett J, Herzenberg LA (1969). Cell sorting: automated separation of mammalian cells as a function of intracellular fluorescence. Science.

[B33] Walters MC, Fiering S, Bouhassira EE, Scalzo D, Goeke S, Magis W, Garrick D, Whitelaw E, Martin DI (1999). The chicken beta-globin 5'HS4 boundary element blocks enhancer-mediated suppression of silencing. Mol Cell Biol.

