# Supplementary figures and images for: A combination of transposable elements and magnetic cell sorting provides a very efficient transgenesis system for chicken primary erythroid progenitors
Source: BMC Biotechnol. 2009 Sep 18;9:81. doi: 10.1186/1472-6750-9-81 (PMC2753566; doi:10.1186/1472-6750-9-81)

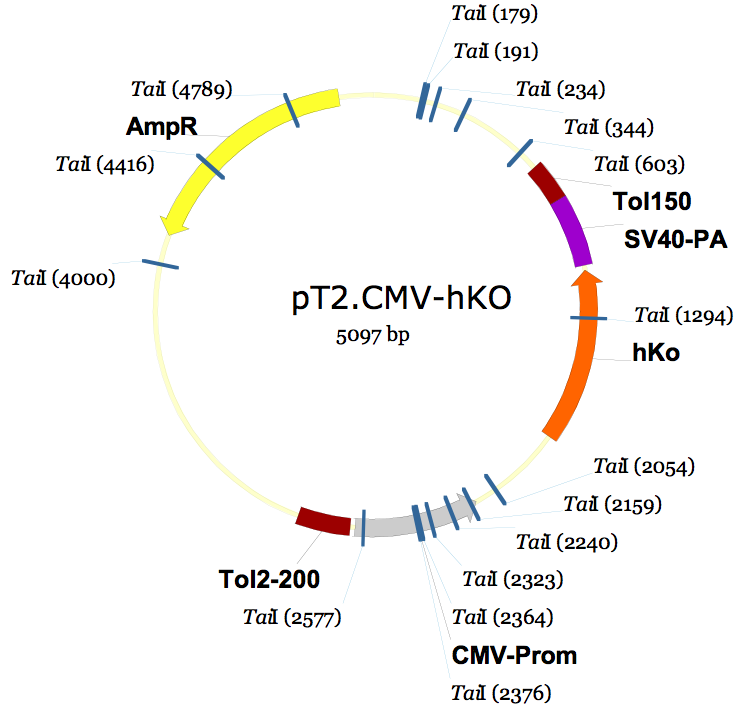

Supplement: Additional file 1 — pT2.CMV-hKO plasmid map. TaiI restriction sites are shown. [file 1472-6750-9-81-S1.PNG]

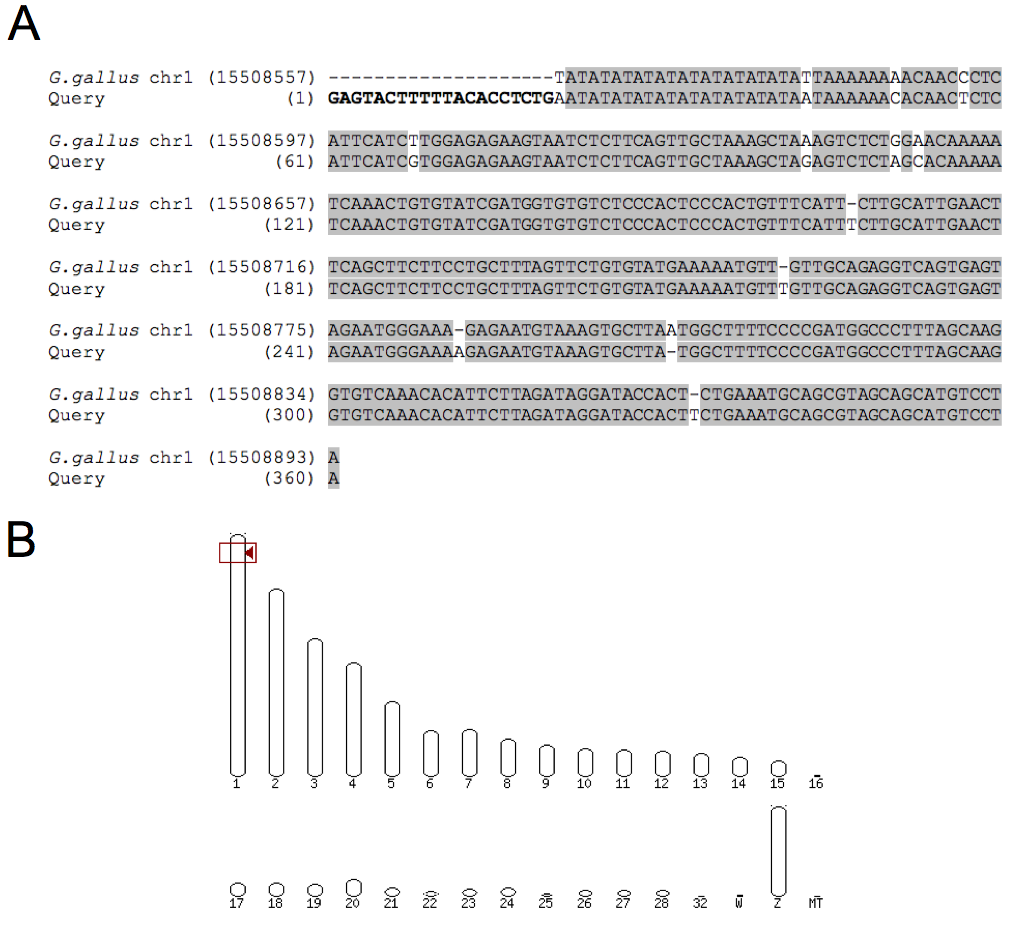

Supplement: Additional file 2 — Identification of the Tol2 construct genomic insertion sites on T2EC clone #2. (A) Alignment of the splinkerette PCR product with the Gallus gallus genome. The sequence part of the Tol2 construct is shown in bold. (B) Localization of the Tol2 construct insertion site into chromosome 1 of the Gallus gallus genome. [file 1472-6750-9-81-S2.PNG]

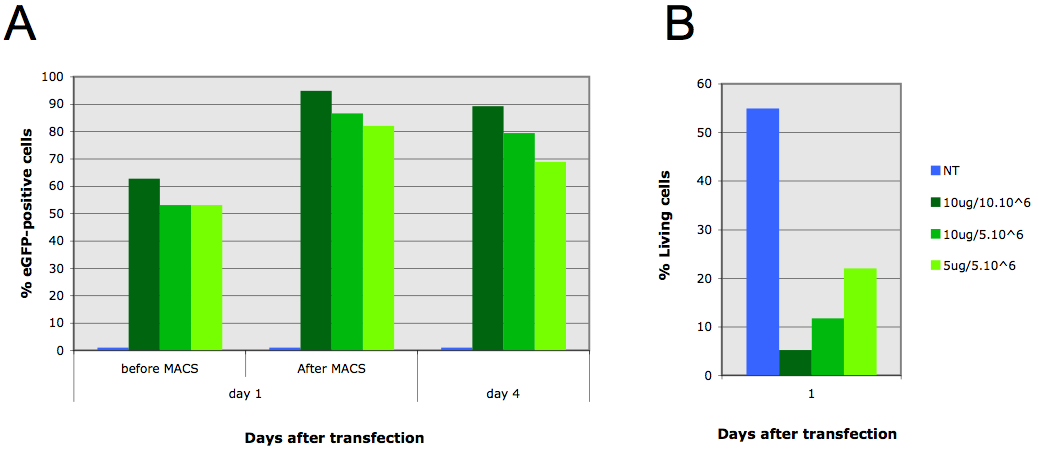

Supplement: Additional file 3 — Influence of the amount of DNA and the cell number on MACS efficiency. (A) Relative proportion evolution, with respect to time after transfection, of eGFP-positive cells within a population of T2EC cotransfected with the eGFP expression pT2MIK-eGFP plasmid and the transposase expression pCAGGS-T2TP plasmid with a 5/1 molecular ratio. Different total DNA (pT2MIK-eGFP+ pCAGGS-T2TP) amounts (5 μg or 10 μg) and cell numbers (5 × 10^6^ or 10 × 106) were tested. Cell fluorescence was analyzed by flow cytometry (FACS). The positive fluorescence threshold is fixed in order to have 99% of the negative cells (i.e. cells transfected with the empty plasmid) below this threshold. A 1% value is, hence, considered as null. (B) Percentage of living cells, for the same conditions as (A), according to the morphology characteristics (size and granularity) measured by flow cytometry (FACS) one day after transfection. [file 1472-6750-9-81-S3.PNG]

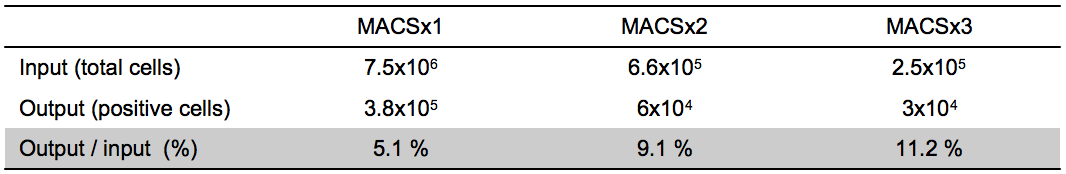

Supplement: Additional file 4 — Cell number before and after each MACS step. A population of T2EC cotransfected with the eGFP expression plasmid and the transposase-supplier helper plasmid (molecular ratio: 1/1) was successively sorted three times on day 1, day 4 and day 7, with respect to time after transfection (Respectively MACSx1 and MACSx2, MACSx3). The cell number was determined before (input) and after (output) each MACS step and the ratio output/input was calculated. [file 1472-6750-9-81-S4.PNG]
